# Response to “Lack of association between bovine leukemia virus and breast cancer in Chinese patients”

**DOI:** 10.1186/s13058-017-0808-7

**Published:** 2017-03-07

**Authors:** Gertrude Case Buehring

**Affiliations:** Division of Infectious Diseases and Vaccinology, School of Public Health, University of California, 16 Barker Hall, Berkeley, CA 94720-7354 USA

We are pleased that our publication [1] inspired the authors to investigate the relationship of bovine leukemia virus (BLV) and breast cancer in Chinese women. However, aspects of the methodology presented cast doubt on the accuracy of the results. The authors state (third paragraph) “Similarly, the commercial ELISA kit used in our study could readily detect BLV-specific antibodies in all cows which were also positive for BLV by PCR, but failed to detect reactive antibodies in Chinese women with or without breast cancer.” Six commercial BLV antibody testing kits are available for veterinary use from the following companies: VMRD, IDEXX, Svanova, Zoetis, Demeditec, and Biovet. Zhang et al. used a kit from BioVet (personal communication with one of the authors). The commercial kits were intended for cattle and were standardized and extensively tested for the level of anti-BLV antibody usually found in BLV-infected cattle, which is much higher than that found in humans [2, 3]. Therefore, it would be unlikely that the veterinary kits would detect the lower level of human anti-BLV antibody that we were able to detect only with immunblotting, and not by ELISA [3]. The commercial kits are not intended for testing human sera; some would be negative for all human sera because the final detection step involves a labeled antibody to bovine, not human, immunoglobulin. Kit directions explicitly say “for veterinary use only” or “intended for detection of BLV antibodies in cattle serum and milk.”

In addition, the PCR methodology is difficult to evaluate. The second paragraph states “In this study, we conducted seven PCRs according to Buehring’s group [3, 4] and one published real-time PCR.” Our reported research [1, 4] used in situ PCR, which requires sample preparation, cycling times/temperatures, reaction mix composition, and a thermal cycler different from those used for standard solution PCR and real-time PCR. It is impossible from the information given to evaluate whether the authors’ in situ PCR techniques were equivalent to what we used. Understandably, the authors were probably confined by the journal’s word limit for a “Letter” and could not give additional information.

It is plausible that the reported lack of detectable BLV infection in Chinese women with breast cancer could relate to lower consumption of bovine food products in China compared to the USA. However, this conclusion cannot be firmly made based on the methodology presented in this publication; further research on this topic is needed.

## Abbreviations

BLV: Bovine leukemia virus
ELISA: Enzyme-linked immunosorbent assay
PCR: Polymerase chain reaction
